# Assistant Personal Robot (APR): Conception and Application of a Tele-Operated Assisted Living Robot

**DOI:** 10.3390/s16050610

**Published:** 2016-04-28

**Authors:** Eduard Clotet, Dani Martínez, Javier Moreno, Marcel Tresanchez, Jordi Palacín

**Affiliations:** Department of Computer Science and Industrial Engineering, Universitat de Lleida, Jaume II, 69, 25001 Lleida, Spain; eclotet@diei.udl.cat (E.C.); dmartinez@diei.udl.cat (D.M.); jmoreno@diei.udl.cat (J.M.); mtresanchez@diei.udl.cat (M.T.)

**Keywords:** Assistant Personal Robot, telecontrol, mobile robot, peer-to-peer video, assisted living

## Abstract

This paper presents the technical description, mechanical design, electronic components, software implementation and possible applications of a tele-operated mobile robot designed as an assisted living tool. This robotic concept has been named Assistant Personal Robot (or APR for short) and has been designed as a remotely telecontrolled robotic platform built to provide social and assistive services to elderly people and those with impaired mobility. The APR features a fast high-mobility motion system adapted for tele-operation in plain indoor areas, which incorporates a high-priority collision avoidance procedure. This paper presents the mechanical architecture, electrical fundaments and software implementation required in order to develop the main functionalities of an assistive robot. The APR uses a tablet in order to implement the basic peer-to-peer videoconference and tele-operation control combined with a tactile graphic user interface. The paper also presents the development of some applications proposed in the framework of an assisted living robot.

## 1. Introduction

The Assistant Personal Robot (or APR) is proposed as a remotely telecontrolled mobile robotic platform with videoconference capabilities. The APR is designed to provide telepresence services [1] primarily for the elderly and those with mobility impairments. Reports from diverse institutions, such as the United Nations [2] and World Health Organization [3], postulate that the proportion of people aged 60 or over is expected to rise from 12% to 21% during the next 35 years as a result of a clear increase of human life expectancy. According to these predictions, elderly care is one of the fields where new technologies have to be applied in the form of passive monitoring systems [4,5] and also as autonomous and tele-operated robots. Such robots can be used to provide assistance to the elderly and people with mobility problems in homes and institutions [6].

The development of assistive robots can provide benefits through the development of optimized solutions for some specific problems that appear in typical household domains [7,8]. A review of the technical description of some mobile robots proposed for telepresence applications is available in [8]. As examples of these applications: in [9] a robotic assistant was used in order to help the residents of a geriatric institution perform their daily routine; in [10] an assistive robot was proposed to help elderly people with mild cognitive impairments; in [7] a telepresence robot showed positive results in domestic elder care assistance; in [11] a telepresence robot was used to improve communication and interaction between people with dementia and their relatives; finally, in [12] a home care monitoring system was proposed, based on a fixed sensor network infrastructure and some physiological sensors, offering the possibility of a virtual visit via a tele-operated mobile robot.

In the context of this paper, a typical example for the application of a household robot is the development of functionalities focused on detecting human inactivity, falls or other accidental situations. In this case, one of the goals is the reduction of the reaction time for external assistance. This aspect is very important because approximately one third of the population aged over 65 falls every year [13] and, in most of the cases, are unable to recover by their own means or ask for assistance [14]. The consequences for elderly people are not limited to physical injuries because a loss of self-confidence also affects mobility drastically [15]. Therefore, telecontrolled mobile robots can be used for social interaction by decreasing isolation and promoting positive emotions and psychological reinforcement.

**Figure 1:**
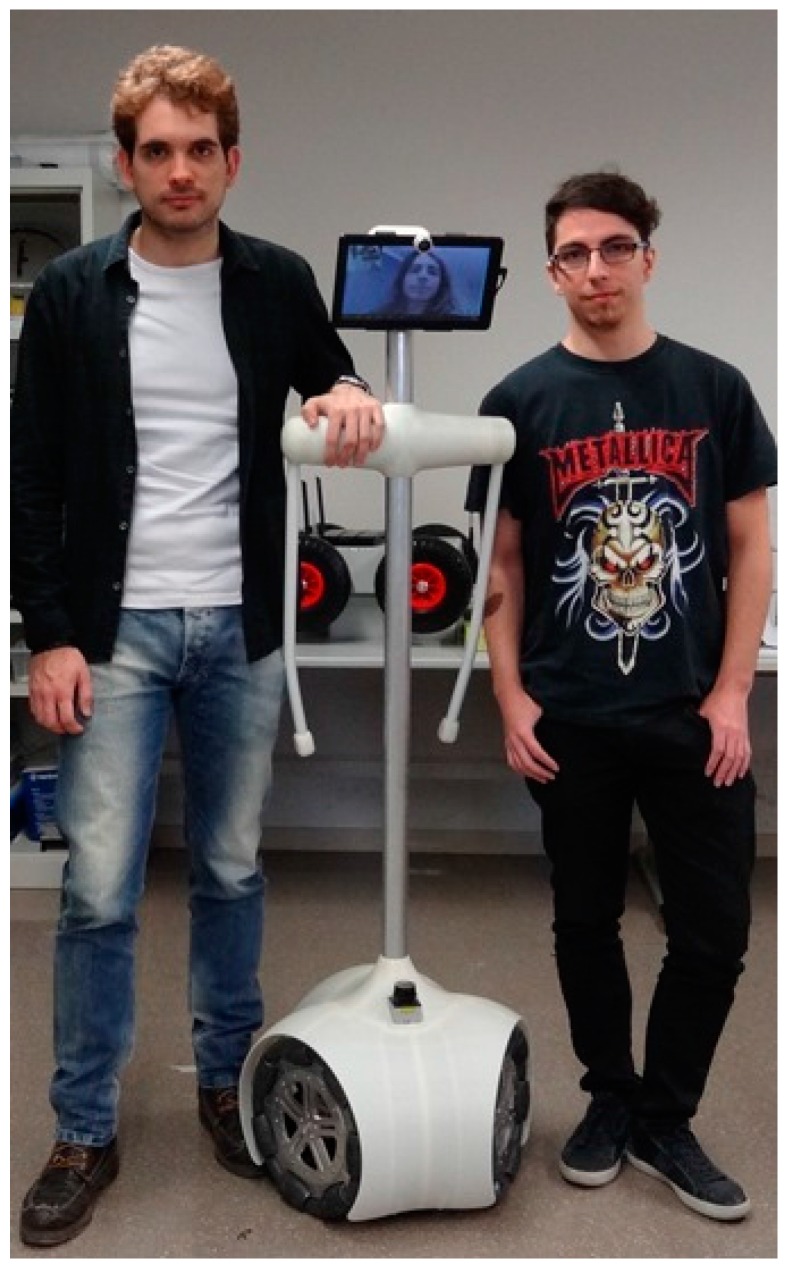
Comparative image of the APR.

The APR is designed to provide remote general assistance and help to elderly people and those with impaired mobility in conventional unstructured environments. Figure 1 shows an image of the APR next to some volunteers to indicate its size. Mobility is provided by omnidirectional wheels that can move the APR in any direction without performing intermediate trajectory maneuvers. The head of the APR is based on an Android Tablet, can be rotated horizontally and vertically, and implements the videoconference capabilities for telecontrol and telepresence. The APR includes onboard Light Detection and Ranging (LIDAR) sensors which detect the distance to the surrounding objects on a two-dimensional plane by measuring the time-of-flight of a rotating laser beam. The main electronic board of the APR uses the information of the LIDAR sensors directly to implement a high-priority collision avoidance procedure which is able to stop the mobile robot. The APR also has two mobile arms with one degree of freedom and which can be rotated clockwise and counterclockwise through 360° and placed at any desired angular orientation. The arms can be used for gestural interaction or as a soft handle as a walking support.

The new contribution of this paper is the complete technical description of a tele-operated mobile robot designed for assisted living. Comparing the capabilities of the APR with the 16 commercial and prototype mobile robots reviewed in [8], the APR includes common devices and functionalities available in other mobile robots designed for telepresence. The APR is not designed with adjustable height, a capability included in four of the 16 robots reviewed. The APR includes an omnidirectional motion system, which is included in two of the 16 robots reviewed. The APR includes ambient sensors, a capability not included in any of the mobile robots reviewed. Finally, the APR includes soft arms that can be used as a support for walking, a feature which is not usually available in telepresence mobile robots.

## 2. Mechanical Design

The mechanical design of the APR is steered by the aim to provide personal assistance services in households or institutions without interfering with the inhabitants. The overall weight of the APR is 30 kg. All the heavy elements in the APR are in the base, close to ground level to ensure a lower center of gravity and stable displacement. The definitive height of the APR was determined by performing an ergonomic test with volunteers of different ages. In this test, the volunteers agreed on a robot height of 1.70 m to ensure a good view of the screen included in the head of the APR. The width was limited to 40 cm to simplify the remote telecontrol of the mobile robot when passing through doorways. The head of the APR contains a panoramic screen that can be moved with two degrees of freedom (up/down and left/right). The torso of the APR has two shoulders with one degree of freedom in order to move the arms forward and backward. The motion of the APR is accomplished with three omnidirectional wheels. The physical design of the APR is inspired in and includes several resemblances to humans in order to operate, maneuver and move the head and the arms in a similar way in a typical household or institutional scenario. In [1], a low velocity was considered a drawback when moving long distances with a telecontrolled mobile robot. This drawback could potentially deter people from utilizing this kind of robots. The APR has a maximum forward velocity of 1.3 m/s, which is comparable with the speed of a person and considered adequate for long indoor displacements, such as along a corridor.

**Figure 2:**
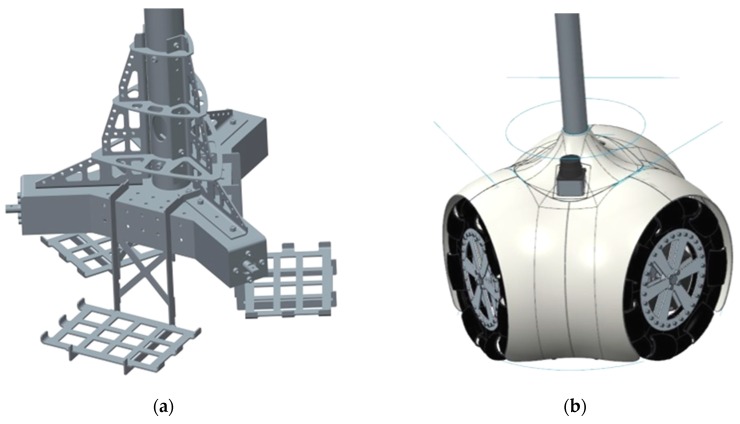
CAD design of the base of the APR: (**a**) mechanical structure and (**b**) plastic cover.

The mechanical structure of the base of the APR (Figure 2a) is made of stainless steel for durability and resistance. This mechanical structure supports three battery shafts for three 12Ah sealed lead acid batteries, three P205 DC motors from Micromotor (Micromotor, Verderio, Italy) and a sloping surface to hold the LIDAR sensors, the main electronic control board and the battery charger system. The APR has three omnidirectional wheels (Figure 1 and Figure 2b) shifted 120° and attached to the motors by conical mechanical connectors. This motion system maintains the engine axle, provides proper torque transmission, and simplifies the assembly of other internal components.

**Figure 3:**
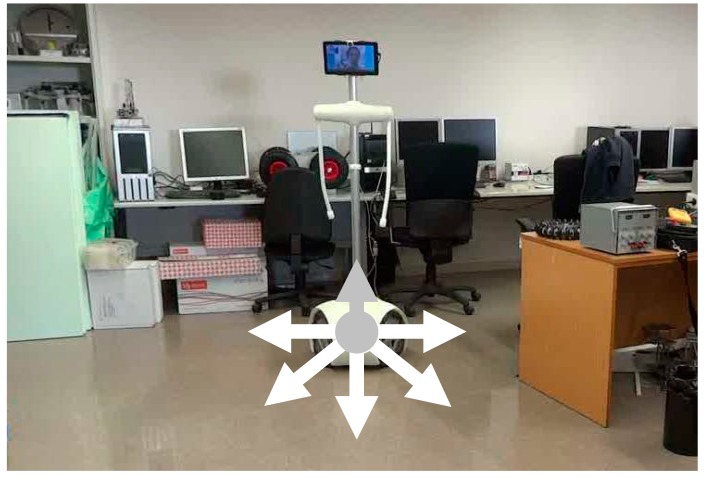
Representation of the direct motion capabilities of the APR.

The omnidirectional wheels contain multiple rollers on the external edge of each wheel creating a holonomic mobile platform where each wheel has two degrees of freedom, allowing wheel spin and perpendicular displacements from the wheel’s forward direction and thus direct displacement in any desired direction (Figure 3). The external white cover of the base is made of ABS plastic (Figure 2b) using a fast prototyping 3D printer. This provides a cheap method of fabrication, a smooth-finished appearance and easy replacement of damaged parts. The circular outline of the base of the robot provides an external space-efficient shape and the absence of protruding and potentially harmful elements. This circular design also minimizes the probability of it becoming accidentally hooked on such furnishings as mats, curtains or clothing.

The chest and shoulders of the APR are located at a height of approximately 1.3 m, slightly lower than the average for human shoulders. This position was chosen to enable elderly people to hold the arms of the robot directly. The shoulders of the APR contain two heavy Micromotor DC motors, which are connected to two soft arms with a 35 cm separation between them. The arms are 55 cm long, just for aesthetical reasons, and can be used as a support by elder people when walking or for gesture interaction. The arms are moved periodically to mimic the natural movements of humans when the APR moves forward.

**Figure 4:**
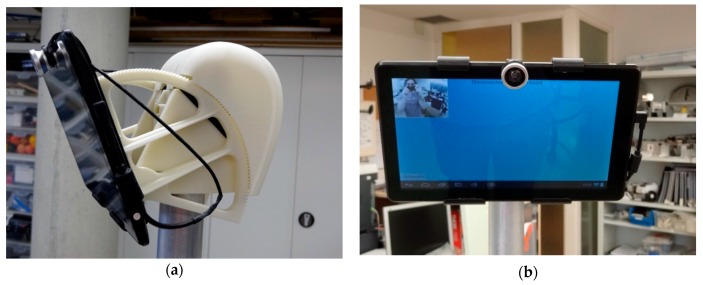
Detail of the head of the APR: (**a**) lateral view, (**b**) frontal view.

The head of the APR (Figure 4) is designed as the main interface between the tele-operator, the mobile robot and the user. The head is based on the Cheesecake 10.1″ XL QUAD tablet (Approx Iberia, Gelves, Spain). This has four processors, a ten-finger touch interface, network connectivity, and multimedia capabilities in order to create and reproduce audio and video in videoconference communication between the APR and a remote tele-operator. The head of the APR has two small Micromotor DC motors (BS138F-2s.12.608) that provide two degrees of freedom. The head can rotate 120° (60° to each side) and also tilt from 0° to 90°: 0° is with the camera pointing at the ground to offer a zenithal view of people lying down and the contour of the mobile robot, while 90° is the case with the camera pointed to the front. The APR is complemented with a low-cost 180° fish eye lens magnetically attached to the camera of the tablet that can provide a panoramic view of the surroundings.

## 3. Electronic Components

**Figure 5:**
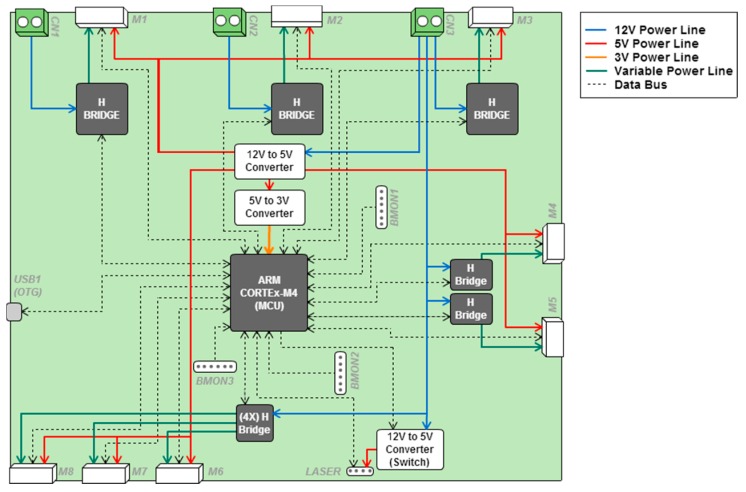
Schematic representation of the motor control board (MCB) of the APR.

The electronic components of the APR are placed at the base and are composed of a motor control board (MCB) and three battery charger modules. The MCB (Figure 5) has an ARM (Cambridge, England) Cortex-M4 based Microcontroller Unit (MCU) with a STM32F407VGT6 processor from STMicroelectronics (Geneva, Switzerland), operating at 168 MHz. This MCB was selected because it is a low power device that is able to control up to seven DC motors with dual-channel encoders while including support for different serial interfaces. This MCU provides a Full Speed (12 Mb/s) USB 2.0 On-The-Go (OTG) wired interface with the tablet device located at the head of the APR. The motors are controlled by four H Bridges on a single Quad-Dual-Bridge Driver integrated circuit (TB6555FLG from Toshiba, Tokyo, Japan), two H Bridges to control the robot’s arms (HIP4020IBZ from Intersil, Milpitas, CA, United States), and three H Bridges to control the motors that drive the omnidirectional wheels (VNH2SP30 from STMicroelectronics). The MCU implements all the specifications of the Android Accessory Development Kit (ADK) so as to be recognized by any Android Tablet or Smartphone through a Full-speed USB wired connection. The MCB communicates directly with different LIDAR sensors (such as the small and compact URG and UTM models, Hokuyo, Osaka, Japan), laser range sensors that provide a two-dimensional planar description of a predefined planar-space around the robot. The URG model obtains new raw data at a sample rate of approximately 100 milliseconds and provides an angle of view of 240° with a maximum operative range of 5.6 m and a maximum current consumption of 0.5 A at 5 V. The UTM model provides a higher sampling rate and operative range but, in this case, the maximum current consumption is 1.0 A at 12 V.

Finally, the MCB is connected to three complementary battery charger boards that provide information about the state of the internal batteries, allowing remote battery management without user intervention. The batteries are charged when the APR is manually plugged in to recharge. Additionally, the tablet of the APR has his own internal battery that is always fully charged in normal APR operation. Thanks to this internal battery, the tablet (and then the audio and video communication capabilities of the APR) remain active and available for calls for several hours after full APR discharge. Table 1 shows the description of the connectors on the MCB (Figure 5).

**Figure 6:**
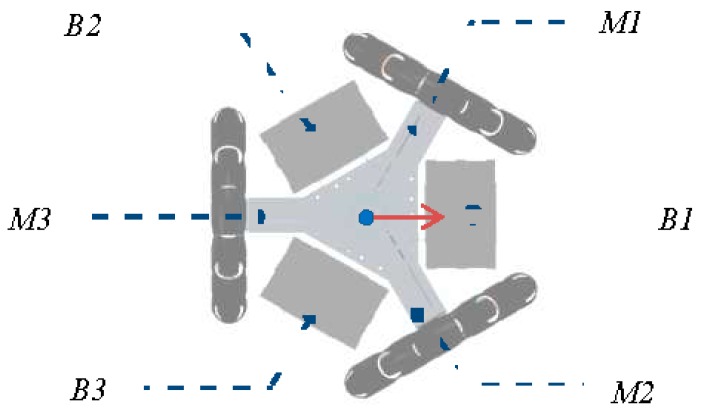
Batteries (B1, B2 and B3) and main DC motors (M1, M2 and M3) at the base of the APR.

The base of the APR (Figure 6) has three high-capacity batteries and three battery charger boards based on an UC3906 Linear Lead-Acid Battery Charger combined with a MJD2955T4 PNP bipolar transistor that regulates the charging process. The three battery charge boards are complemented with a main AC/DC switching adapter (220 V AC/18 V DC) for battery charge and a secondary AC/DC switching adapter (220 V AC/12 V DC) for simultaneous powering the MCB during recharging of the internal batteries.

Table 2 shows the electrical current provided by the batteries of the APR for a set of predefined robot motions. In this prototype, the batteries operate independently in order to power different parts of the APR directly. Battery 1 (Figure 6-B1) is exclusively dedicated to powering motor 1 (Figure 6-M1) and battery 2 (Figure 6-B2), to powering motor 2 (Figure 6-M2). These two motors are used exclusively for forward (and backward) displacement, which is usually performed at full speed and for long periods of time. Battery 3 (Figure 6-B3) is used to power motor 3 (Figure 6-M3), the MCB, the LIDAR sensors, and the Tablet, which also has its own batteries. Table 2 shows that the maximum energy consumption is reached when the APR goes forward or backward at full speed, the rotation and the transversal displacement of the APR is performed with the three motors in simultaneous operation. Most of the current provided by Battery 3 is dedicated to powering the LIDAR sensors. These can be electrically disconnected in order to reduce power consumption when the APR is in standby operation. The APR has been tested in periods of up to four hours but there is currently still no estimation of battery life as this will largely depend on usage.

## 4. Software Implementation

The APR is defined as a remotely operated platform that has to be compatible with environments designed for humans rather than for robots. The APR has to offer a reliable remote control operation with a stable remote videoconference system in order to ensure its application as a tele-operated assisted-living robot. The transmission of audio, video and control orders is carried out by using direct peer-to-peer (P2P) or client-to-client communication to reduce delay in the remote control system.

### 4.1. Transmission of Motion Control Orders

**Figure 7:**
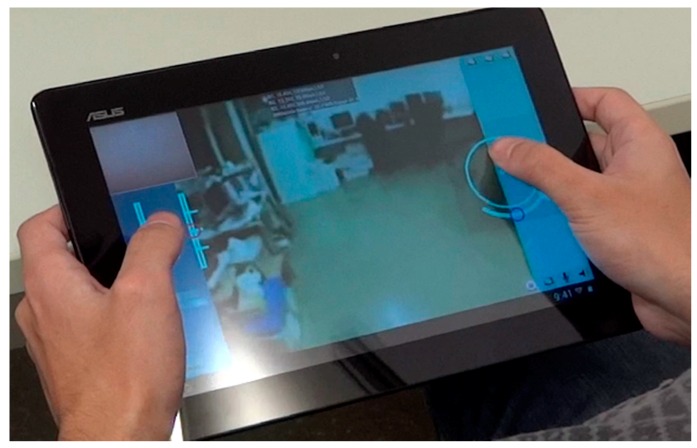
Android APP used for the remote control of the APR.

The remote control of the APR is performed with an Android application (APP) running in a remote smartphone or tablet. Figure 7 shows an image of the remote control interface. This is based on the touch-screen capabilities of such mobile devices. The telecontrol APP is composed of an on-screen joystick that is used to control the position of the robot, two sliders to control the pan and tilt of the robot head, two sliders to control the robot arms (left and right arm), and an extra slider used to rotate the APR over its vertical axis. The telecontrol APP can be used with one or two fingers by trained and non-trained people. The remote control of the mobile robot is not a challenging task because the high-mobility capabilities of the APR do not usually require intermediate trajectory maneuvers. Future implementations of the telecontrol APP will include such additional features as direct control of the trajectory of the APR with small head displacements [16] in order to allow remote robot control by people with impaired mobility.

Table 3 shows the structure of the commands sent in a single seven-byte user datagram protocol (UDP) packet where each byte has a specific interpretation. The main on-screen joystick codifies the desired input motion orders in two bytes: robot angle (Angle) and movement velocity (Module). Other controls, such as the rotation of the APR, the orientation of the head, and the rotation of the arms, are coded and submitted simultaneously. The complete control of the mobile robot is performed with network messages containing only seven bytes.

The UDP protocol provides fast transmission rates at the expense of omitting error detection procedures and also with a lack of knowledge about the status of the communication. The use of the UDP protocol in a real-time remote control system is justified since there is no interest on recovering lost or delayed packets containing outdated information. In this implementation, a control command is sent at least every 100 ms according to the operator’s tactile-gestures. Finally, the APR automatically stops after a time-lapse of 300 ms with no new control commands as a way to avoid the execution of unsupervised movements. At this moment, the APR automatically stops in case of network connection failure and remains inactive waiting for a new connection. In the future, this state will be complemented with the possibility of returning to the starting point or another predefined point.

The tablet located in the head of the APR receives the UDP control packets and generates specific low-level orders for the MCB through the Fast USB wired connection. Table 4 show the basic instruction set orders interpreted by the MCB. There are specific motion orders (starting with MF and MB) that are used for long straight forward and backward displacements. These displacements require the application of an electric brake to the unused (and unpowered) M3 motor to prevent free rotation and the continuous rectification of the angle of the wheel.

### 4.2. Transmission of Audio and Video Streaming

The bidirectional transmission of audio and video data streaming is a fundamental task in a mobile robot designed for tele-operation and remote interaction. This section presents a detailed description of the generic bidirectional audio and video transmission system developed for the APR. This system is specifically designed to connect two Android devices: the tablet that controls the APR and the remote operator’s smartphone or tablet. 

#### 4.2.1. Videoconference Architecture

**Figure 8:**
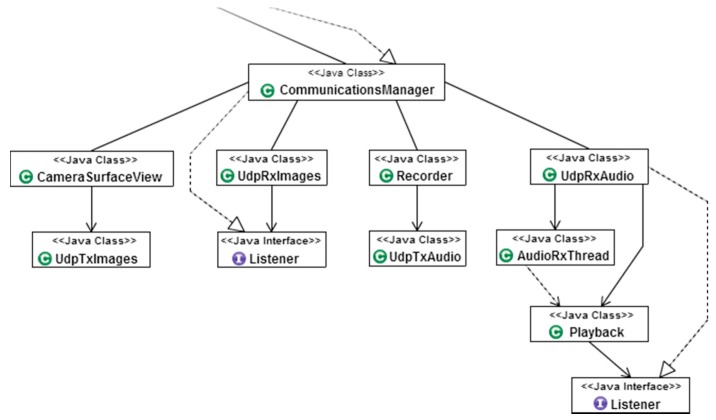
Depiction of the videoconference system hierarchy.

The videoconference system defined in an Android device is controlled from the upper-class *CommunicationsManager*. This class contains all the methods required to configure and initiate a new videoconference with a remote device. The videoconference system is made up of four different classes: *UdpRxImages*, *CameraSurfaceView*, *Recorder*, and *UdpRxAudio*. Each of these classes is executed in an independent thread and an independent network socket to limit the possibility of blocking the device in case of unexpected network problems when performing long operations. Figure 8 shows the hierarchy of the videoconference system.

The exchange of data between classes is performed though *custom event listeners* (from now on, listeners) and by creating callback functions that are triggered by specific events and notified to all the classes registered to that listener. The main reason that justifies the use of listeners instead of handlers is that listeners are immediately executed once received, while handler callbacks are queued for future execution.

#### 4.2.2. Network Communications Protocol

The audio and video data is exchanged by using UDP packets based on sending single packets to a specific address and port instead of creating a dedicated communication channel. Since UDP does not perform any control on the packets, it offers a time efficient communication protocol. This lack of control makes UDP one of the best transmission methods for communications where delivery time is more important than data integrity. The main reason of using UDP in audio/video communications is the prevention of communication delays caused by errors produced in a single packet. Then, the lost data packets are ignored and communication is restored when the next packet is received. Unlike the video and audio data, the control parameters for the communication are simultaneously transmitted by using the transmission control protocol (TCP). The use of TCP sockets provide more reliable communication channels, which are used to exchange essential configuration data from videoconference parameters that each device has to know in order to successfully decode the video and audio data received from the remote device.

#### 4.2.3. Video Communications

Video transmission/reception is an indispensable feature for a tele-operated assisted living robot. Video communication is a computationally expensive application that needs to be efficient, reliable, robust, and with low-delay in the transmissions.

Currently, one of the most commonly used encoders to perform live streams is the H.264/MPEG-4 due to its high-compression capabilities. This encoder is designed to generate stable and continuous transmissions with low-bandwidth requirements but has several drawbacks. On one hand, this encoder can have a variable communication delay from 3 to 5 s and high-motion scenes can appear blurred because of the dependence between consecutive frames [17]. On the other hand, this encoder requires highly optimized libraries which are difficult to integrate into a custom application. The bidirectional video communication method implemented in the APR overcomes these drawbacks by transmitting video as a sequence of compressed images in JPEG format instead of using a common video streaming format. This approach simplifies the implementation and control of the communication. Therefore, the bidirectional video communication implemented in the APR has to acquire, compress and submit surrounding images while receiving, decompressing and showing the images submitted by the remote device.

The procedure for image acquisition in the Android environment starts in the *CameraSurfaceView* class, which extends the Android class *SurfaceView*. The *SurfaceView* class is used as a canvas providing a dedicated drawing area where the preview of the camera is displayed. Once the camera has been initialized, the *onPreviewFrame(byte[] data, Camera camera)* callback is triggered each time a new image is obtained, retrieving an array containing the YUV image data encoded with the NV21 Android native format for camera preview as well as the camera instance that has captured the image. The images are then compressed to the standard JPEG format (which reduces the file size at the expense of quality and computing time) to be submitted as an image packet. Table 5 shows an example of typical common image resolutions with the relationship between size, quality and time required for the JPEG compression of the image, and the theoretical maximum rate of frames per second (fps) that can be obtained in communication with the Tablet of the APR. Video communication can use any resolution available in the telecontrol device. Under normal conditions, the time required to compress one image is similar to the time needed to decompress the image. The large size reduction achieved with the JPEG compression (Table 5) is because the original YUV-NV21 image format is already a compressed image format with averaged and grouped U and V color planes so this image has less spatial variability than a conventional raw RGB color image and can be described with less information and size after compression.

The implementation of a videoconference system requires image acquisition, image compression, transmission, reception, and image decompression in a bidirectional way. The real frame rate of the image acquisition devices is usually 15 fps. Some images may be lost during this process: (a) because a new image is available while the previous image is still being compressed so this must be discarded; (b) because of UDP transmission on the network; and (c) because a new image has been received while the previous image is still being decompressed so this must be discarded. However, current smartphones and tablets usually include multiple CPUs and use different isolated threads for transmission and reception so they can achieve higher videoconference frame rates than when only one CPU is available for processing.

**Figure 9:**
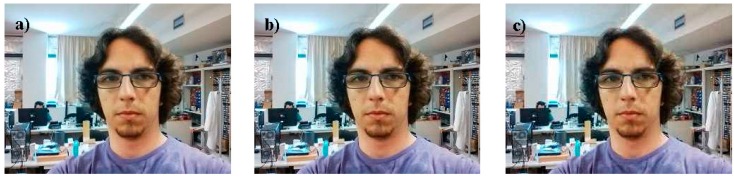
Sample 320 × 240 color image showing the small differences obtained after a JPEG compression and decompression procedure with quality settings of (**a**) 100%; (**b**) 60%; (**c**) 30%.

Figure 9 shows a visual example of the impact of the quality parameter on a JPEG compression and decompression. Figure 9 shows the face the remote operator, acquired with a smartphone, compressed as JPEG, submitted to the network, received by the tablet of the APR, decompressed, and shown on the screen of the APR for visual interaction. In this example with small images, the difference between 100% and 60% qualities is almost imperceptible to the human eye while the image size is 12 times smaller. There are also small differences between 60% and 30% qualities in terms of compressed image size, processing time and expected frames per second. The APR uses a video transmission procedure configured to operate with a starting default resolution of 320 × 240 pixels with a JPEG quality factor of 60%. Then, the expected average image submitted to the network is 56 times smaller than the original YUV-NV21 images acquired by the device, and 84 times smaller than the RGB version of the original image. This image resolution can be changed manually during the videoconference or adapted dynamically to the network bandwidth capabilities.

The compressed image is sent to the *UdpTxImages* thread, which creates a new UDP packet containing the image. Once an image has been received at the *UdpTxImages* thread, a flag is activated to notify the *CameraSurfaceView* class that there is a transmission in progress, so newly generated images are not transmitted. This flag is used to indicate to the *CameraSurfaceView* that there is no need to compress and send new images to the *UdpTxImages* thread until the current transmission is completed. The main goal of this procedure is to avoid wasting CPU cycles by compressing images that will not be sent. However, this method slightly increases the video delay since the next image to be transmitted is not compressed until the last transmission ends. In general, a video transmission of compressed JPEG images of 320 × 240 pixels obtained at a frame rate of 15 fps and compressed with a quality factor of 60% with an average size of 2720 bytes requires a network bandwidth of 0.318 Mb/s per video streaming (0.881 Mb/s for a color image of 640 × 480 pixels). This image-based video streaming gives shorter delays in communication but may require higher network bandwidth than alternative buffered-based streaming systems. In general, typical domestic networks have enough upload and download bandwidth for two-directional video streaming communication with color images 640 × 480-pixels processed at 15 fps.

Video reception is carried out by the *UdpRxImages* execution thread. Once initialized, this thread starts receiving UDP packets containing the frames transmitted by the remote device. Each time an image packet is received, the data is decoded into a bitmap using the Android method *BitmapFactory.decodeByteArray()*. The new bitmap is then sent to the application main activity using the *OnImageReceived()* listener. Once the new image has been received at the main activity, the interface view is updated by the User Interface (UI) thread. In order to improve the memory usage at the reception side of the application, the *inMutable* flag from the *BitmapFactory* class is set to true, forcing the *BitmapFactory* class to always use the same memory space to store new decoded images by avoiding the creation of a new bitmap each time a new image is received. When using the *inMutable* flag the bitmap used to decode the received image must have the same resolution. If an image with a different resolution is received, the bitmap memory used by the *BitmapFactory* class is reallocated using the new image dimensions, so the only situation where a new bitmap is created is when the image resolution of the images is changed.

There is no a specific optimum resolution suitable for all situations and networks. The resolution of the local and remote images used in the video communication can be changed dynamically. If required, the video communication sends a TCP packet to the remote device to request a list containing the resolutions at which the remote camera can operate. In order to obtain such information, the *getSupportedRes()* function from the *CameraSurfaceView* class is called. This function uses the *getParameters()* method from the *camera* class that contains the instance of the camera that is being used. The *getSupportedPreviewSizes()* method from camera instance parameters retrieves a list of the supported resolutions for live video preview. This list is then sent back to the device that initially made the query and all the resolutions are shown on the screen. This system allows a fast change or selection of the best image resolution required for each specific situation.

Once the image resolution has been changed, a new TCP packet containing the new image width and height is sent to the remote device. When this packet is received, the remote device calls the *changeResolution()* method from the *CameraSurfaceView* class. This method stores the new resolution and enables a flag that notifies the *onPreviewFrame()* callback to change the configuration of the camera. In this case, the camera is stopped and the method *setNewConfiguration()* is called in order to change the video configuration and restarts the video transmission.

#### 4.2.4. Audio Communications

The transmission of audio during a remote interaction with the APR is a natural, direct and efficient communication channel between the person in charge of the telecontrol of the robot and the person assisted by the robot. The main problem that arises when establishing bidirectional audio communication is the delay between audio transmission and reproduction at the remote device when using buffered-based libraries such as VoIP/SIP. This problem is produced because the Android VoIP requirements force the application to ensure that a minimum amount of data is received (and buffered) before starting the audio playback. This audio transmission is similar as the P2P procedure used for video transmission. This implementation uses the Android *AudioRecord* and *AudioTrack* classes to record and reproduce audio data in both devices respectively (telecontrol device and APR head device). Although *AudioRecord* is not designed to perform audio streaming, the functionality of this class has been modified to generate small audio chunks that can be sent through the network immediately and reproduced at the remote device.

Firstly an instance of the *AudioTrack* class is initialized with the following specifications: Sample rate of 8000 Hz, encoding format PCM 16bit, 1 channel (mono). Once initialized, the *AudioRecorder* instance stores all the data obtained by the device microphone in an internal buffer. When the internal buffer is filled with 3840 bytes of voice data, the buffer is sent to the *UdpAudioTx* thread, wrapped inside an UDP packet, and transmitted to the remote device. The UDP packet is received at the *UdpAudioRx* execution thread of the remote device and sent to the *AudioTrack* class running inside the *Playback* thread. This system has been initialized with the same audio parameters as the *AudioRecord* instance and it queues the received audio data for reproduction. In order to avoid an increasing delay on the reception side of the application, packets received with a time difference greater than 50 ms, or empty audio streams, are dropped thus ensuring that the delay of a single packet does not accumulate into a whole communication delay.

### 4.3. External Network Server

The communication of the APR with a remote telecontrol device not located in the same local area network (LAN) is a specific problem that requires the development of an external network server. The main function of this server is to provide a common control space where telecontrol clients and robots are managed in order to establish a P2P communication between two devices. In this common space, the server is continuously waiting for a new incoming connection. This is then associated with an event listener executed in an independent execution thread. Once a new thread has been started, the server requests the mandatory information related to the connection itself (Table 6). The communication can be initiated by the APR or by a remote telecontrol device according to a predefined policy usage.

The connection data is analyzed to determine if the device is connected under the same network domain as the server. This procedure is realized by checking the domain of the IP address provided during the connection process. If the IP address provided exists inside the private address ranges (Table 7), the device is then under the same network domain as the server and thus the internal IP address will be used to send packets to the device. If the device fails at providing the requested information the communication will be closed, otherwise, the communication will be accepted and registered to the server. Once a device is connected, the specified role determines the accessible functions for the device. Therefore, when an operator wants to initiate the control of a specific APR, a packet containing the robot name is sent to the server and, once received; the server sends a request to both devices to fire multiple UDP packets to the server through specific ports. The objective of these UDP packets is to open a direct path between the client and the robot on both network firewalls by using the UDP hole punching technique [18,19]. This protocol allows direct P2P communication between different registered APRs and different registered telecontrol devices using the ports opened when starting the communication with the external server. When P2P communication starts, the server closes the UDP communications and uses the TCP socket to send and receive specific packets from both devices. As a final task, the external server keeps an updated list of on-line APRs ready for new connections.

In general, the use of an external server allows long-range internet communications between a client and a robot regardless of whether they are connected at the same network domain or not. However, when using UDP packets this method can fail to engage a communication channel if any of the devices is connected to the internet using mobile networks (such as 3G or 4G) in the case of staying under multiple levels of the network address translation (NAT) protocol, see [19] for additional details. This problem can be solved by using TCP packets instead of UDP packets or by changing the configuration of the communication channel in order to accept UDP packets.

## 5. Tests

This section presents the preliminary usability tests performed with the APR and also the specific test of the high-priority collision system implemented in the mobile robot. Future works will center on developing usability tests focused on the experience of the users such as the one proposed in [1] and on improving the human-robot interaction experience according to the guidelines proposed in [20].

### 5.1. Preliminary Usability Test

The preliminary usability tests were performed with 12 volunteers aged from 12 to 50 years during prototype development in order to evaluate the degree of satisfaction of the different features implemented in the APR according to the different classification: “poor”, “acceptable” and “good”. Future exhaustive tests will use additional classification levels such as proposed in [1]. The results obtained in these preliminary tests have been used to refine the design and implementation of the APR.

The first test consisted of introducing the robot to each of the 12 volunteers, who were then invited to rate the appearance of the APR. The volunteers gave an average score of “good”, showing that the participants liked the APR design. Future in depth usability tests will be performed with target users in order to evaluate the usability of the APR, but currently, the focus was to obtain the subjective impression caused by the design of the mobile robot. The assumption was that a good impression is required to facilitate the continuous use a mobile robot at home.

The second test consisted of evaluating the videoconference capabilities implemented in the APR and the remote telecontrol APP under static conditions. During this test, the 12 volunteers were grouped into pairs and placed in different rooms in the same building and network. Each volunteer participating in the experiment played both roles: as a robot tele-operator and as a passive user, performing a total of 12 experiments. The volunteers rated this static videoconference as “acceptable” and “good”. Some of the volunteers complained about a small but sometimes noticeable desynchronization between the audio and the images in the video communication. The cause of this desynchronization is that the audio and images of the video are sent in different network UDP packets that may not be received with the same cadence as they are submitted at. This problem was partially improved by including synchronization information into the audio packets to allow automatic discarding of delayed audio packets with no audio information.

The third experiment consisted of evaluating the maneuverability of the APR. In this test each of the 12 volunteers has to control the APR as a remote tele-operator. Each volunteer was trained for ten minutes in the use of the remote telecontrol APP and then the APR had to be displaced between different starting points and destinations. The volunteers rated this telecontrol as “good”, expressing increasing confidence during the experiment.

The last test consisted of evaluating the videoconference capabilities implemented in the APR and the remote telecontrol APP under dynamic conditions. During this test, the 12 volunteers were grouped into pairs. Each volunteer participating in the experiment played both roles: as a static robot tele-operator and as a passive user who has to walk along a corridor with the mobile robot, performing a total of 12 experiments. This test was performed in the same building and network. The volunteers rated this dynamic videoconference as “poor” and “acceptable”. The cause of this low score was a loud background noise from a mechanical vibration generated in the APR during displacements. This problem was addressed and partially solved by adding rubber bands to the structure that supports the tablet to minimize the transmission of mechanical vibrations to the microphone embedded in the tablet. Then, this dynamic experiment was repeated with these improvements and the volunteers rated the videoconference as “acceptable”.

### 5.2. High-Priority Collision Avoidance System

The APR is a tele-operated mobile robot designed to operate in domestic environments with limited space and its operation must be compatible with the presence of static objects such as walls and furniture, and also dynamic objects such as people and doors [21]. In general, control of a tele-operated robot is strongly conditioned by external factors such as network stability and the operator’s experience.

**Figure 10:**
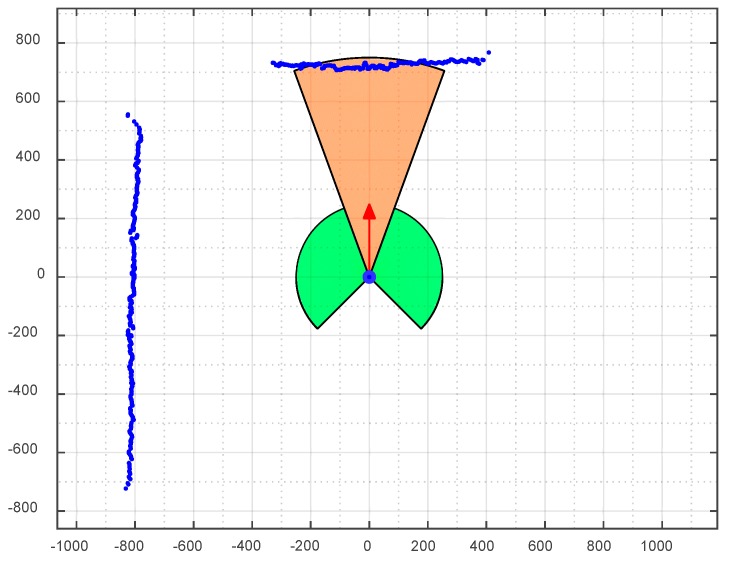
Depiction of the APR position and direction (red arrow), distance data points detected with the LIDAR (blue points) and depiction of the frontal (brownish) and lateral (greenish) safety areas around the mobile robot.

The APR has a maximum forward velocity of 1.3 m/s and, for this reason, includes a high-priority collision avoidance system implemented in the MCB. This high-priority procedure processes the information from the LIDARs directly in order to stop the mobile robot automatically without delay when a collision situation is estimated or detected regardless of any other consideration. Figure 10 depicts the planar information gathered by the main LIDAR placed parallel to the ground at a height of 38 cm. The high-priority procedure is based on the delimitation of different semicircular safety areas around the APR [22]. Currently, other alternatives, such as an automatic change in the trajectory [23], are not implemented as they may cause confusion to the operator in charge of the telecontrol of the mobile robot.

The collision avoidance system automatically stops the APR if the LIDAR detects anything inside one of the different safety areas. In this emergency situation, the tele-operator of the APR can rotate the robot or perform backward or transversal displacements. The radius and orientation of the safety areas around the APR are adjusted dynamically according to the trajectory and velocity of the mobile robot. For example, the APR stops if it tries to pass through a doorway at maximum speed while it will not stop when passing at a reduced speed.

**Figure 11:**
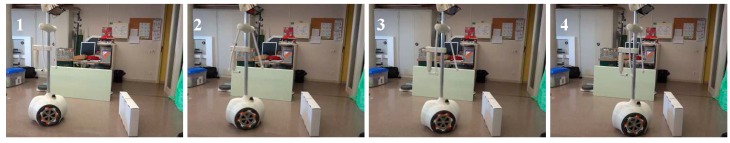
Sequence of images (**1**–**4**) obtained in a collision avoidance experiment with the APR moving forward.

A set of different experiments were carried out to verify the dynamic definition of the safety areas around the APR. Figure 11 shows a sequence of images while conducting one experiment where the APR goes forward at full speed in the direction of an obstacle. The depiction in Figure 10 corresponds approximately to sequence 3 in Figure 11 in which an object is detected in front of the mobile robot. The results of different collision avoidance experiments have shown that the APR can avoid collisions except in the case of objects with transparent glasses or mirrors because these objects are not properly detected by laser LIDAR sensors.

Table 8 summarizes the collision avoidance experiments performed. In each experiment, the APR is configured to go forward at a fixed speed. The first column in Table 8 shows the forward speed relative to the maximum forward speed of 1.3 m/s. The second column in Table 8 shows the radius of the frontal safety area (see Figure 10) which is fixed according to the forward speed. The last column shows the final distance APR-obstacle measured when the robot has been effectively stopped. Table 8 shows the proposed calibration between the robot velocity and radius of the safety frontal distance, which has a value of 120 cm when the APR reaches the maximum forward velocity of 1.3 m/s. The minimum gap-distance between the APR and the object after braking at full speed was 22.5 cm. The procedure for stopping the mobile robot is based on using the motors as electrical brakes by forcing a shortcut through the H-bridges.

In the future, this high-priority collision avoidance system will be improved in order to estimate the trajectory of dynamic obstacles around the mobile robot and reduce the braking distance.

## 6. Applications of the APR

This section proposes some sample applications for the APR to take advantage of its capabilities as a tele-operated assisted living robot.

### 6.1. Mobile Videoconference Service

**Figure 12:**
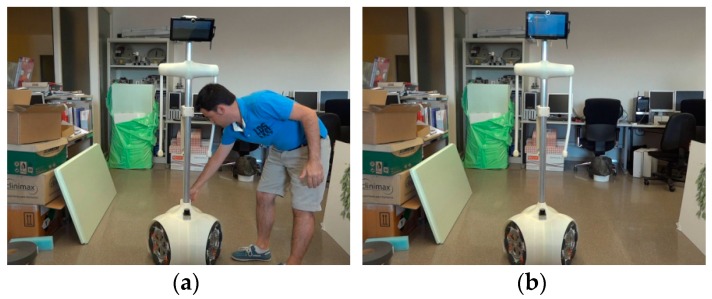
(**a**) Starting the APR; (**b**) APR initializing the onboard sensors and initializing the Tablet of the head.

The APR has been designed as a telecontrolled mobile videoconference service prepared to provide social and assistive services to elderly people and persons with impaired mobility. In the case of this application, the APR is located at home and the mobile robot can be configured to automatically to accept calls from registered remote operators or services that will take the control of the mobile robot during a mobile videoconference. The APR can be turned on by pressing a single button (Figure 12) so as to make it easy for users with low technological skills to use. 

This unique button starts the MCB which configures its main USB as a host interface. The tablet is also configured to start automatically when activity is detected in a USB host interface. The tablet of the APR always operates as a slave device connected to the USB host interface of the MCB. Once started, the tablet of the APR automatically launches the client APR application, which also connects to a global server to register the robot as an available remotely operated APR ready for a new connection (Figure 12). The APR only requires specific operational configuration the first time it is started (global server IP and robot name), and after that, the robot remembers the parameters used for the configuration and uses them as its default parameters.

On one hand, the APR operates like a basic phone or videoconference device that can use the information of the onboard LIDAR sensors automatically to generate a call or an alarm for relatives and preconfigured registered services. This call can also be generated simply by touching the screen of the tablet. On the other hand, the people or services registered can use the telecontrol APR APP to initiate a remote communication with the APR as audio only, audio and video, or audio and video combined with remote telecontrol operation. The remote tele-operation APP establishes a connection with a global external server and shows a list of all the authorized and available APRs registered for this service.

**Figure 13:**
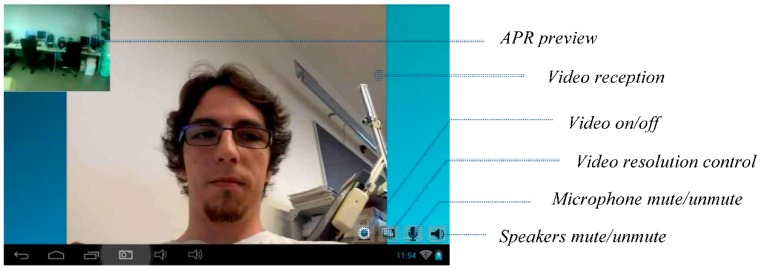
Screenshot of the Tablet of the APR showing the face of the person calling.

Figure 13 shows the screen of the tablet of the APR with a small window that previews the local image acquired by the APR and submitted during the videoconference, the image received during the videoconference and four main buttons used to control the communication. The buttons enable or disable the submission of video, the submission of audio, the reception of audio, and also change the resolution of the images used in the videoconference. Currently, the complete videoconference starts automatically but the automatic response of the APR can be configured depending on the authorization level of the service in charge of the call.

One of the objectives of the APR is to enable direct bidirectional communication with relatives and technical or medical services to be established. On one hand, this communication can be used to generate social interaction with elderly people. On the other hand, the sensors on the APR can detect problems or inactivity and automatically generate alarms. In such a context, the APR can be used by a remote tele-operated service to explore a typical household scenario visually, evaluate the situation and reduce the reaction time if external assistance is needed.

### 6.2. Mobile Telepresence Service

**Figure 14:**
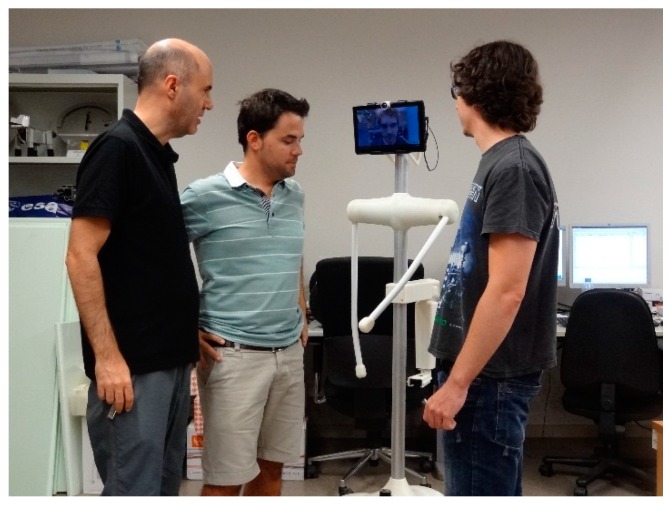
Depiction of the telepresence capabilities of the APR.

Alternatively, the concept of telepresence [8] implemented in the APR can be used directly by people with impaired mobility [24] located in their home in order to connect with another place. In the case of this application, the APR is located at one facility and the mobile robot is configured to accept calls from registered users who will take control of the mobile robot as telepresence service. Figure 14 shows a simulated example of the APR used as a telepresence platform during an informal talk between three colleagues. In this simulated case, the APR is only used as a tool for social interaction. This is an alternative application demanded by people with impaired mobility and which will be deployed in future work.

This application will be installed in a typical industry, office or institution to offer telepresence contact between employees. Telepresence is achieved by offering a physical and mobile depiction of the person who is controlling the APR. The telecontrol application of the APR enables direct control of the robot’s arms and head as a way of simulating the creation of non-verbal communication between groups of people who usually collaborate with each other. In the future, this non-verbal communication will be enhanced with the inclusion of configurable buttons with predefined and personalized combination of small arm and head movements.

Future work based on this application will focus on deploying the APR as a telepresence service for the industry as an informal way of maintaining contact between groups of employees in case of someone having impaired mobility that impedes their presence and interaction with the group. The hypothesis is that frequent and informal telepresence contact can improve productivity while contributing to reducing the effort spent on unpleasant, long or difficult journeys. In this context, the APR will be tested as a telepresence tool that may contribute to enhancing the employability of people with impaired mobility.

**Figure 15:**
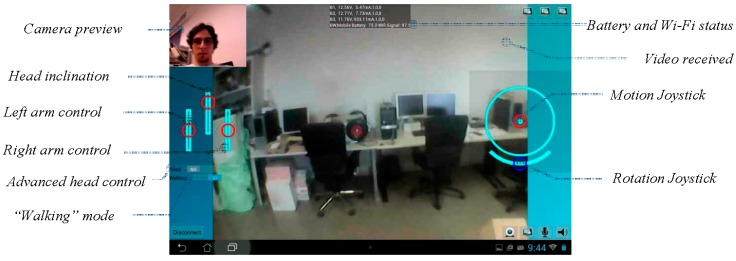
Screenshot of the remote telecontrol application.

Figure 15 shows a screenshot of the telecontrol application of the APR. This application is used by a trained operator and the interface is operated with one or two fingers. The interface includes a circular motion joystick where the red circle depicts the current direction of the APR and a rotation joystick used specifically to rotate the mobile robot. The external circle of the motion joystick is also used to show the relative location of obstacle warnings detected by the APR. There are additional sliders to control the head and the arms of the robot. This screen shows the status of the battery and wireless communication as text messages at top center of the screen but this information will be replaced with small graphic indicators in the future.

### 6.3. Walking Assistant Tool

**Figure 16:**
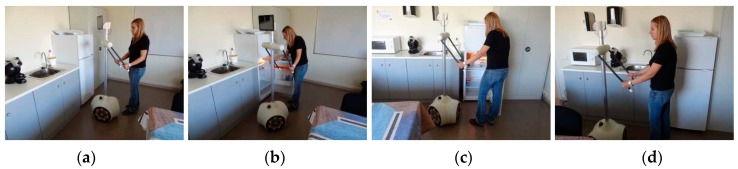
Applications (**a**–**d**) of the APR as a support for walking.

The APR can be used as a walking assistant tool [25] and to avoid the use of additional devices in limited spaces [26]. Figure 16 shows a sample image of a person using the soft arms of the APR as a walking support. In this mode, the velocity of the APR is automatically limited while the arms are set at an angle of 35° to the back. The functionality of the mobile robot as a walking support can be remotely supervised by using the front or rear camera of the tablet and by rotating the head. The orientation of the motors of the arms has a closed control loop and any disturbance (force applied by the user) [26] can be used to refine the displacement of the robot automatically. Finally, in this mode, the information gathered by the LIDAR sensors is used automatically to center the robot when passing through doorways or along a narrow corridor.

### 6.4. Scheduling Tool

**Figure 17:**
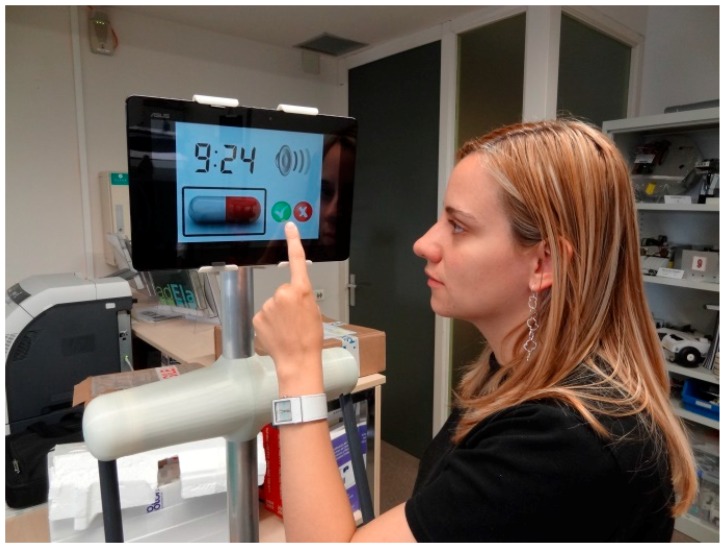
Simulation of the APR providing a daily or regular routine notification.

The APR can be used to help elderly people to remember their daily routines [27,28]. This task is performed by means of individual programmable alarms that show a visual just-in-time representation of a daily routine on the screen of the APR, combined with additional acoustic signals or recorded advices. In this application, the tactile screen of the tablet of APR is used for direct feedback. Figure 17 shows a simulation of the APR operating as a scheduling tool. In our region, there is a public service for elderly people. This involves a daily call to remind them of all the daily routines but the hypothesis is that a visual indication at the scheduled moment of, for example, taking a medicine will be more effective than a daily phone-call.

### 6.5. Fall Detection Tool

**Figure 18:**
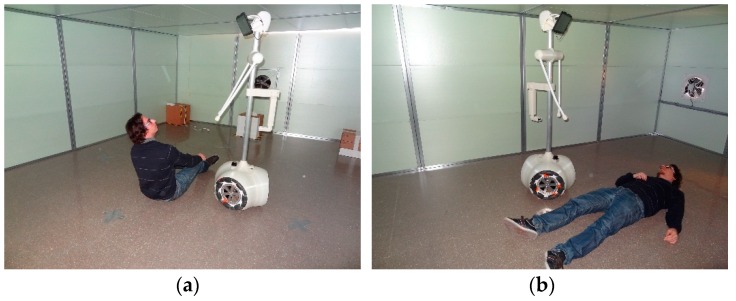
Simulation of the APR used as a fall detector (**a**) with activity and (**b**) without activity.

In this context, the last APR application proposed in this paper is the detection of falls or inactivity [4,5]. This functionality requires a sequential analysis of the raw data provided by the onboard LIDAR sensors, the detection of the background objects and abnormal user movements. Figure 18 shows a simulation of the APR used as a fall detector that generates a warning when the volume of the user detected in the information provided by the LIDARs decreases or increases suddenly. Then, an automatic call is generated if the user does not press a button that appears on the screen of the tablet on the APR.

### 6.6. Mobile Ambient Monitoring Platform

The APR can be used as a mobile ambient monitoring platform that gathers information from the environment. For example, in [29], a mobile robot with specialized onboard sensors was used to measure such environmental parameters as air velocity and gas concentrations in the air. Other ambient parameters, such as temperature, humidity, and luminance, were also measured using low-cost sensors. These environmental values are directly correlated with living conditions and were used to detected areas or rooms with abnormal conditions automatically. Alternatively, ambient information can be gathered with a fixed-sensor network infrastructure. For example, the GiraffPlus project [12] allowed remote examination of body temperature, blood pressure and electrical usage based on a sensor network infrastructure and some physiological sensors. This system offers the possibility of a virtual visit via a tele-operated robot to discuss the physiological data and activities occurring over a period of time and the automatic generation of alarms in case of detecting instantaneous problems or warnings based on long-term trend analysis.

**Figure 19:**
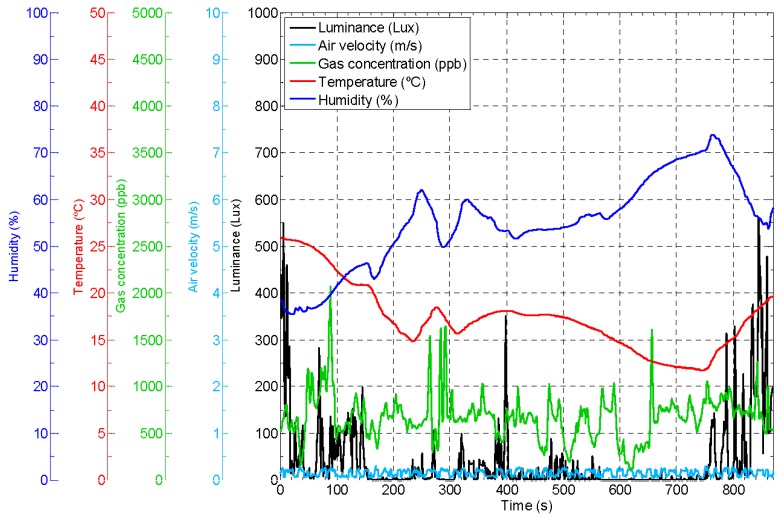
Example of ambient information automatically gathered by the APR.

Figure 19 shows the ambient information gathered by the APR during a telecontrol experiment based on the methodologies presented in [29,30] with a common sampling sensory time of 1 s. Figure 19 show the temperature registered by the APR with extreme values of 26 °C in one room and 12 °C in another room with an open window (in winter). The humidity reached abnormal values when the APR was in the room with an open window. The luminance level was poor with the lowest values in an indoor corridor. The air velocity was always low and probably originated by the displacement of the APR. Finally, the measurement of volatile gas concentrations in the air can be performed by using one sensor per gas type or by the use of a versatile sensor [29], which can be configured to measure different gasses. In this case, a photo-ionization sensor (PID) was configured to measure acetone because this was a substance used in the area explored [30]. The results showed a low concentration of acetone in the air during the experiment. The APR can be used as a supporting tool to implement a mobile ambient monitoring platform and develop new assisted-living applications.

## 7. Conclusions

This paper presents the conception and application of a tele-operated mobile robot as an assisted living tool. The proposed Assistant Personal Robot (APR) is described in terms of mechanical design, electronics and software implementation. The APR uses three omnidirectional wheels in order to offer high-mobility capabilities for effective indoor navigation. The APR has a maximum speed of 1.3 m/s and implements a high-priority collision avoidance system in the electronic board that controls the motors of the mobile robot, which can stop the APR regardless of the current motion orders. The APR is described in terms of applications proposed to develop the concept of a tele-operated assisted living tool mainly focused on assistive applications for elderly people and those with impaired mobility. The preliminary experiments performed in this paper have been used to refine the design of the APR as a tele-operated assisted living robot. Future work will focus on improving the design of the APR and the development of the assistive services proposed in this paper.

## Figures and Tables

**Table 1:** Description of the MCB connectors.

TAG	Description	TAG	Description
***CN1***	Connector for battery 1.	***M6***	Connector for the Head Pan motor.
***CN2***	Connector for battery 2.	***M7***	Connector for the Head Tilt motor.
***CN3***	Connector for battery 3.	***M8***	Unused.
***M1***	Connector for the front left wheel motor.	***USB1***	Micro-USB “On the Go” connector.
***M2***	Connector for the front right wheel motor.	***LASER***	Connector for a Hokuyo LIDAR device
***M3***	Connector for the back wheel motor.	***BMON1***	Connector to monitor battery 1.
***M4***	Connector for the Left Arm motor.	***BMON2***	Connector to monitor battery 2.
***M5***	Connector for the Right Arm motor.	***BMON3***	Connector to monitor battery 3.

**Table 2:** Average electrical current of the batteries when performing different predefined motions.

Motion	Battery 1 (mA)	Battery 2 (mA)	Battery 3 (mA)	Total (mA)
**Standby**	5	5	480	490
**Stopped**	5	5	890	900
**Go Forward**	700	803	1202	2705
**Go Backward**	934	951	880	2765
**Rotate (left/right)**	120	97	976	1193
**Move right**	176	194	1582	1952
**Move left**	154	181	1447	1782

**Table 3:** Structure of an UDP motors control packet.

ID	Head Pan	Head Tilt	Left Arm	Right Arm	Module	Angle	Rotation
**Byte**	0	1	2	3	4	5	6

**Table 4:** Instruction set orders interpreted by the MCB.

Command	Parameters	Description
AL *d*\r	*d*: from −45 to 45°	Moves the left arm to the specified position in degrees.
AR *d*\r	*d*: from −45 to 45°	Moves the right arm to the specified position in degrees.
HP *d*\r	*d*: from −60 to 60°	Rotates the head of the robot to the specified position in degrees.
HT *d*\r	*d*: from 0 to 90°	Tilts the head of the robot to the specified position in degrees.
M *S1S2S3*\r	*S1*: PWM from 0 to 60% *S2*: PWM from 0 to 20% *S3*: PWM from 0 to 20%	Fixes the Pulse Width Modulation (PWM) applied to motors M1, M2 and M3 of the APR.
MF *S*\r	*S*: PWM from 0 to 60%	Applies electric braking to M3 and fixes the same PWM to M1 and M2 to generate a forward displacement.
MB *S*\r	*S*: PWM from 0 to 60%	Applies electric braking to M3 and fixes the same PWM to M1 and M2 to generate a backward displacement.
TL *S*\r	*S*: PWM from 0 to 20%	Fixes the same PWM to M1, M2 and M3 to rotate the robot to the left.
TR *S*\r	*S*: PWM from 0 to 20%	Fixes the same PWM to M1, M2 and M3 to rotate the robot to the right.

**Table 5:** Relationship between image size and JPEG quality.

Color Image			JPEG Compression			
Resolution (Pixels W × H)	RGB Size (Bytes)	YUV-NV21 Size (Bytes)	JPEG Quality (%)	JPEG Size (Bytes)	Compression Time (ms)	Fps (max)
**176 × 144**	76,032	50,688	30	1233	12.46	80
**176 × 144**	76,032	50,688	60	1391	11.74	85
**176 × 144**	76,032	50,688	100	11,911	16.50	60
**320 × 240**	230,400	153,600	30	2280	19.96	50
**320 × 240**	230,400	153,600	60	2718	19.51	51
**320 × 240**	230,400	153,600	100	33,617	24.74	40
**640 × 480**	921,600	614,400	30	6351	29.90	33
**640 × 480**	921,600	614,400	60	7526	30.26	33
**640 × 480**	921,600	614,400	100	115,491	55.14	18
**720 × 480**	1,036,800	691,200	30	7618	32.54	30
**720 × 480**	1,036,800	691,200	60	9316	31.86	31
**720 × 480**	1,036,800	691,200	100	126,430	61.68	16
**1280 × 720**	2,764,800	1,843,200	30	17,810	72.47	13
**1280 × 720**	2,764,800	1,843,200	60	21,230	72.87	13
**1280 × 720**	2,764,800	1,843,200	100	311,409	131.72	7
**1280 × 960**	3,686,400	2,457,600	30	23,699	96.58	10
**1280 × 960**	3,686,400	2,457,600	60	28,647	98.02	10
**1280 × 960**	3,686,400	2,457,600	100	426,045	177.25	5

**Table 6:** Required data to connect with the external server.

Tag	Type	Provided by	Description
Role	String	Device APP	A string containing the role of the device connected to the server (service or robot)
Name	String	Configuration	A string containing the name of the robot or service. If no name is specified, the string “Default” is used as a name.
NAT Port	Integer	Network protocol	Network Address Translation (NAT) port assigned to the communication
IP Address	InetAddress	Network protocol	The IP address of the device that requested the connection (external IP)
Local IP Address	String	Device APP	The IP address of the device that requested the connection (internal IP)

**Table 7:** Ranges of private IP addresses.

RFC1918 Name	First Available IP	Last Available IP
24-bit block	10.0.0.0	10.255.255.255
20-bit block	172.16.0.0	172.31.255.255
16-bit block	192.168.0.0	192.168.255.255

**Table 8:** Collision avoidance test results.

Relative APR Forward Speed (%)	Radius of the Frontal Safety Area (cm)	Distance APR-Obstacle When Stopped (cm)
100	120.0	22.5
88	108.6	23.0
75	96.4	26.3
66	86.7	25.5
50	72.3	30.8
33	56.3	29.0
25	48.9	30.2
